# The Influence of Target Properties on the Accuracy of Reflectorless Distance Measurements

**DOI:** 10.3390/s21196421

**Published:** 2021-09-26

**Authors:** Joanna Gmitrowicz-Iwan, Magdalena Myszura, Tomasz Olenderek, Sławomir Ligęza, Heronim Olenderek

**Affiliations:** 1 Institute of Soil Science, Environment Engineering and Management, University of Life Sciences in Lublin, Leszczyńskiego St. 7, 20-069 Lublin, Poland; joanna.gmitrowicz@gmail.com (J.G.-I.); slawomir.ligeza@up.lublin.pl (S.L.); heronim_olenderek@sggw.edu.pl (H.O.); 2 Institute of Forest Sciences, Warsaw University of Life Sciences, Nowoursynowska St. 159, 02-776 Warszawa, Poland

**Keywords:** reflectorless electronic distance measurement, reflecting surface, total station, density

## Abstract

Recent years have brought dynamic developments in surveying equipment and techniques. These include reflectorless electromagnetic distance measurement (RL EDM), which is used in a range of devices, especially total stations. Studies concerning the influence of the reflecting surface on the accuracy of RL EDM tend to focus on the colour of the measurement surface, while the influence of the density and thickness of materials is usually neglected. Therefore, this study undertook to examine 53 samples representing various materials of dissimilar features: colour, type of surface and density. The results show that dark and mat surfaces cause higher RL EDM errors than bright, gloss materials. Nonetheless, 76% of the results were in compliance with equipment specifications. Moreover, it was found that the density of the samples had significant impact on the overall accuracy. RL EDM to EPS (expanded polystyrene sheets, low-density material, commonly called Styrofoam) involved a significantly higher error rate. It demonstrates that total station measurements and laser scanning should be performed cautiously, especially with regard to materials of low density (e.g., EPS) and on short distances, where the value of relative error is high.

## 1. Introduction

Electronic distance measurements (EDM) are widely used in many areas, thus exceeding the boundaries of classical geodesy [1,2,3,4,5,6]. Therefore, science is trying to expand the knowledge on the factors influencing the accuracy of the measurement type in question. Agents disturbing EDM can be categorised into three groups: the instrument setup, the measurement path and the measurement target. The first group consist of technical parameters of the instrument, the stability of the support etc. The properties of the measurement path include the distance of measurement and atmospheric conditions [7,8]. The last component is the target. Using the prism ensures the highest accuracy of the measurements, while the results of reflectorless (RL) EDMs exhibit higher variability [9,10,11]. The accuracy of RL EDM can change depending on the target’s parameters, such as material, density, chemical composition, thickness, surface roughness, colour, transparency, temperature and angle, which can contribute to scattering, refraction and reflection of the laser beam [12,13].

The first component affecting the EDM is the instrument setup. It includes technical parameters of the device, for example measuring range or laser wavelength. It is vital that the stability of the setup is ensured. Moreover, when the instrument is placed in the measurement chamber (to protect it from environmental conditions), the laser beam distortion by the glass window should be taken into account [10,14].

Additionally, the EDM accuracy is influenced by the path of the beam. The distance could affect the measurement. In general, the larger the distance, the larger the error [15]. Another component is weather conditions, i.e., air temperature, pressure, humidity etc. In certain cases, accurate results could be obtained only after applying atmospheric correction factors [9,10,16].

The changes in RL EDM accuracy caused by the target is widely investigated. One of the basic aspects is the surface colour, examined by instrument manufacturers and scientists alike. Materials of different colours have different albedo, which is the ratio of radiation reflected from a surface to the total radiation incident on the surface [17]. Brightly coloured materials have high albedo, therefore, RL EDM to such targets is more accurate. Moreover, surfaces that have a higher level of the red component, such as white, yellow or red, give better scanning results, since this component corresponds to the colour of the laser beam. Dark colours, namely black, brown, grey and blue, tend to disturb the measurements [15,18].

Another factor is the target surface structure, which could affect the refraction and reflection of the beam. In general, flat gloss surfaces reflect a greater percentage of incident radiation, however, according to Bolkas and Martinez [15] semi-gloss and rough materials could improve the accuracy of RL EDM.

Moreover, RL EDM could be affected by target density, internal structure and thickness. The distance measured to some materials, especially synthetic ones, would be longer than the real value. The laser beam penetrates the material causing measurement disturbance. The greater the target’s thickness, the greater the error [19].

Partially transparent materials are known to reflect only part of the incident beam, and in extreme cases (high transparency) the beam is reflected by the background, thus, falsifying the measurement [13].

Another important factor influencing RL EDMs is the angle between the target surface and the laser beam, which can degrade accuracy. Distance measurements to targets with low angles (not perpendicular to the incident beam) are prone to errors [15,20].

Therefore, it is worth learning more about the parameters which impact the precision of the RL EDM. The purpose of this study was to investigate whether and how the parameters of the target affect the accuracy of RL EDM. Furthermore, we investigated changes in accuracy with regard to low-density materials, using different types of EPS (expanded polystyrene sheets, commonly called Styrofoam), since this topic received little scholarly attention. We aimed at a potential practical application of our results, therefore, investigated materials were those commonly used in construction.

## 2. Materials and Methods

Our research included seven materials commonly used in architecture: various types of wood (polished and untreated boards), synthetic materials, expanded polystyrene sheets (EPS, material commonly called Styrofoam, used in construction), ceramic tiles, metal, brick and paper (Table 1). Due to a potential practical application of this work, we have selected samples of materials most often used in construction and easily available on the market. Samples differed in density, internal structure, surface structure (gloss, mat or coarse), colour and thickness. Materials of such features are popular reflecting surfaces in field geodetic RL EDM. Each specimen was flat and identical in size, 18 × 20 cm. The samples are listed in Table 1.

The results section consists of a few groups. The first section considers the measurement absolute errors on distances 5, 10 and 20 m to all types of tested materials. The second part describes the relative errors. The third section is a comparison of the RL EDMs accuracy to samples of paper, ceramic, plastic, metal, wood and brick of different colours and the same type of surface (glossy). The fourth tested parameter was the type of surface: glossy and matt. The next element of the research was density. We used specimens of EPS (expanded polystyrene sheets, commonly called Styrofoam, in white colour), which differed in density and thickness. The last part is a general comparison of the similarities between the samples.

**Figure 1:**
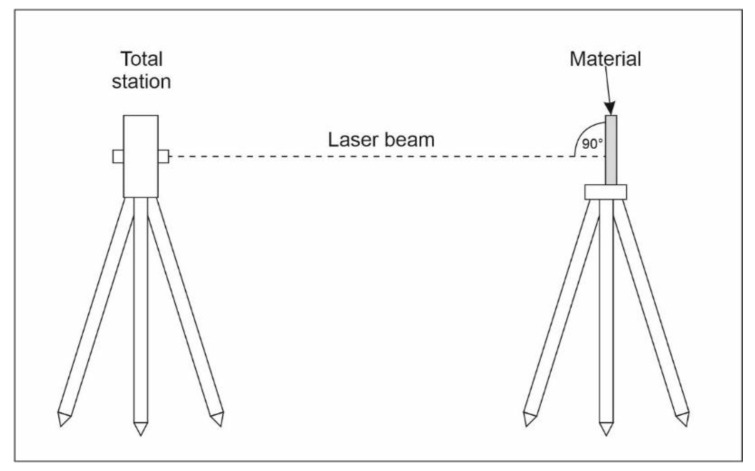
The experimental setup.

The measurements were performed using Topcon ES-105 reflectorless total station. It uses coaxial phase shift measuring system in distance measurements, signal source is red laser diode 690 nm, class 3R. The accuracy according to the manufacturer [21]: ±(2 + 2 ppm × distance in km) using a prism, ±(3 + 2 ppm) mm for reflectorless measurements (white and grey surface on the distance to 100 m). Figure 1 shows an experimental setup. One of the tripods (the right side of Figure 1) was used for material samples, it was fixed during the whole measurements. It was set 10 m from the wall to mitigate the background impact. The background was a flat wall covered with white chalk paint. The samples were placed in a supporting base, which was fastened to the tripod, the central point of the frame in the centre of the tripod. The base retained materials perpendicular to the laser beam. The second tripod (the left side of Figure 1) was used to support the total station, it was fixed during each series of measurements (5 m, 10 m and 20 m). The first and the last measurements of each series were taken using the prism centred on the right-side tripod. This was the baseline measurement. Then the prism was replaced by the material supporting base. The distance measured in reflectorless mode to each material was compared with the distance measured to the prism. The tests were carried out on distances: 5 m, 10 m and 20 m. The RL EDMs were taken in 5 repetitions to each material in the fine measurement mode. The tests were carried out in a laboratory to limit the influence of atmospheric conditions.

The statistical analysis was performed using Statistica 13.1 software (StatSoft Poland, Crocow). It included the mean errors of distance measurements (absolute measurement error: the differences between the reference distance, to the prism, and the distance measured in the reflectorless mode), the relative measurements error, the standard deviation. The normality of distributions was assessed using the Shapiro–Wilk test. The relationships between absolute measurement errors and thickness/density of samples were evaluated by Pearson’s linear correlation test. ANOVA and Tukey a posteriori tests were conducted to determine the significance of differences in measurement accuracy to bright and dark, gloss and mat materials. Additionally, a multivariate technique of cluster analysis was used to determine the similarities between the materials, based on the absolute distance measurement errors, using the single-linkage clustering and Euclidean distance.

## 3. Results and Discussion

### 3.1. Absolute RL EDM Errors

The standard deviations of RL EDM for all samples and distances were low and ranged from 0 to 2 mm, therefore, the standard deviation values for the individual samples are omitted in the tables. In most cases (62%) the absolute error of the distance measurement took a negative value, in the range of −5.8–0.0 mm (Table 2). Similar negative results were shown in the paper by Clark and Robson [22] and Lambrou and Pantazis [20]. The accuracy of distance measurements according to the manufacturer [21] for reflectorless measurements (white and grey surface on the distance to 100 m) was ±(3 + 2 ppm) mm. In 76% of cases, the accuracy was correct and did not exceed this range.

### 3.2. Relative RL EDM Errors

The relative errors of RL EDM were the highest at a distance of 5 m and the lowest at a distance of 20 m—the higher the distance, the lower the relative error (Table 3). This indicates that the type of reflecting surface is most essential at short distances due to the fact that the error of a few millimetres could change the measured distance significantly. In our study, the biggest difference was noted in the case of sample No. 39 (EPS) at a distance of 5 m—it was 0.35%, while at 20 m it was only 0.06%. It could be assumed that at shorter distances the influence would be even higher. Therefore, during geodetic measurements, EPS targets should be avoided. The topic of targets’ density influence, especially those made of polystyrene, require thorough investigation in the future because the existing body of research merely accounts that EPS does cause measurements errors, without providing an explanation.

### 3.3. Colour of the Samples

**Figure 2:**
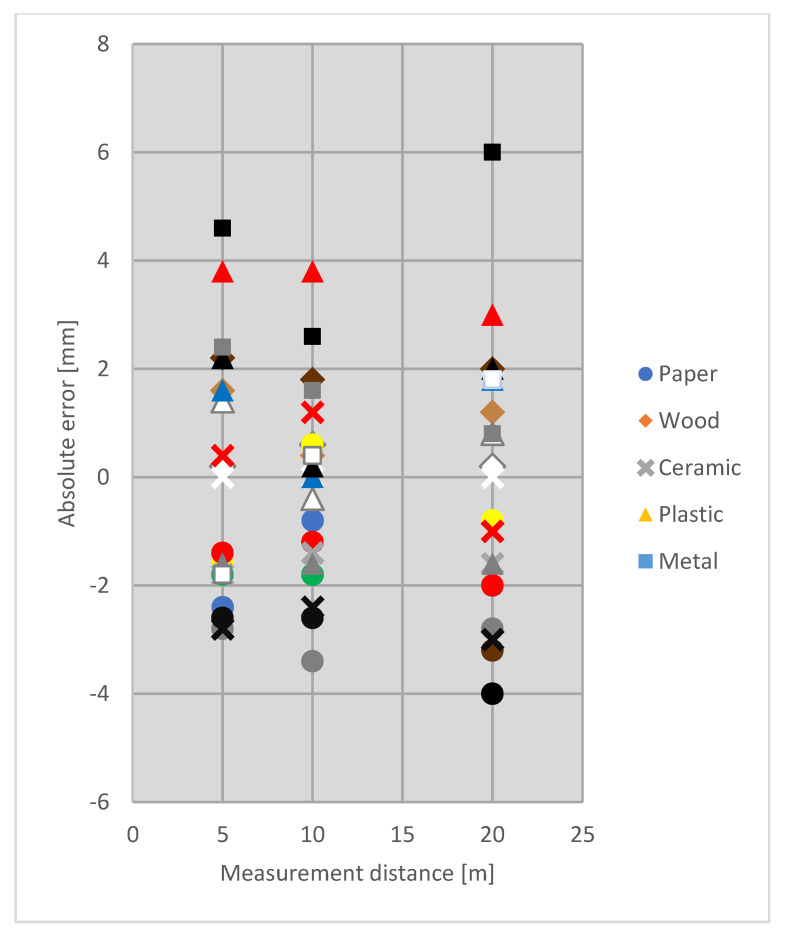
Mean values of absolute RL EDM errors on distances: 5, 10 and 20 m with regard to various materials and colours.

The errors were higher mostly for dark materials (brown, grey, black, Figure 2), which is a common phenomenon [15,18]. Moreover, the errors of RL EDM to dark samples (on average −1.671 mm) were significantly higher than to bright ones (−0.676 mm, the lowest significant difference value was 0.800 mm). Brightly coloured materials have high albedo, therefore, RL EDM to such targets is more accurate. Dark colours, tend to disturb the measurements [15,17,18]. Despite statistically significant values of RL EDM errors to dark and bright samples (excluding samples of EPS) most results (96%) were in compliance with manufacturer specification. The samples which showed the best reflecting properties were ceramic tiles.

### 3.4. Type of Surface

**Figure 3:**
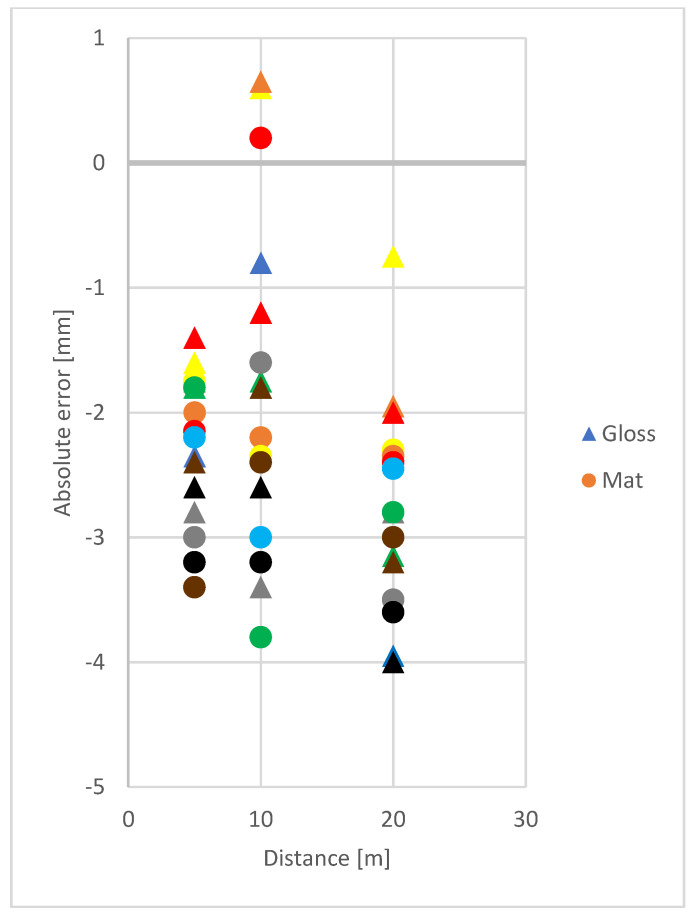
Mean values of absolute RL EDM errors on distances: 5, 10 and 20 m with regard to gloss and mat paper samples in various colours.

The study showed differences in accuracy between gloss and mat paper of the same colour (Figure 3). The average absolute error to gloss targets was −1.467 mm, while to mat targets it was −2.123 mm. The lowest significant difference value was 0.633 mm, therefore, the difference between measurement error to gloss and mat samples was statistically significant. Higher accuracy of RL EDM to gloss targets could be explained by a higher amount of energy returning to the laser scanner, especially in the case when the reflecting surface is oriented orthogonal to the laser beam [15]. However, type of surface, just like surface colour, did not cause errors higher than those given in the instrument specification.

### 3.5. Low-Density Materials

The distance measurement results were highly distorted when targeting the EPS (expanded polystyrene sheets, commonly called Styrofoam) samples, with errors up to 17.6 mm on the distance of 5 m (Table 4). The errors were higher in thick samples (No. 32, 33, 35, 38). The laser beam was not reflected by the surface but spread within the material structure, increasing the distance [19]. It is difficult to compare our results with papers of other authors. Usually, articles provide information only about sample thickness, not density. However, absolute RL EDMs errors, on various distances, were mostly of positive value [13,19,23]. Results similar to ours were obtained by Lenda and Marmol [22]: the value of absolute error on the distance of 5 m was 12.4 mm and 11.1 mm on 15 m. Lambrou and Pantazis, [20] in their research showed that in the distance of 50 m, the absolute error were of negative value. This opposite result could be caused by different parameters of materials used by authors, for example high density of material.

The samples of EPS varied in thickness and density. Results showed that measurement errors depend highly on the sample density, not its thickness, which was confirmed by the analysis of Pearson’s linear correlation coefficients (the significance limit of a correlation coefficient based on Student’s *t*-test, r = 0.707, *p* = 0.05, *n* = 6). The assessment did not reveal any significant relationships between the thickness of the samples and the absolute measurement errors. On the other hand, a strong influence of density was noted at all distances: 5 m (r = 0.806), 10 m (r = −0.811) and 20 m (r = −0.839). The lower the density, the bigger the error.

### 3.6. Similarities between Materials—Cluster Analysis

**Figure 4:**
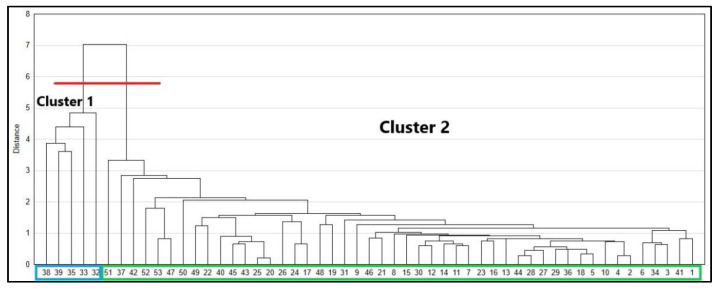
Dendrogram showing clusters of variables on the basis of similarity. 1–53—numbers of samples according to Table 1.

From the cluster analysis, it could be seen which samples were similar according to values of absolute distance measurement errors. The materials were divided into two groups (Figure 4). The first cluster included the specimens exhibiting the most significant difference from the others (38, 39, 35, 33, 32) that is EPS. The binding distances showed a close correlation between the rest of the tested materials (Cluster 2), simultaneously highlighting an influence of material characteristics on measurements: within Cluster 2 smaller units of strong associations were distinguished, mainly based on the type of material (e.g., 45-43, 11-7, 4-2), type of surface (e.g., 25-20, 24-17, 11-7, 4-2), or surface colour (e.g., 24-17, 44-28). The cluster analysis proves that the most important factor, which strongly influences RL EDM, is the density of the reflecting surface.

## 4. Conclusions

Our study shows that:

1. Most of the analysed materials shorten the distance measured in reflectorless mode.
2. The colour and the type of material surface (glass or mat) affect the accuracy of RL EDM, the errors are greater in the case of dark and materials. However, in both cases, the errors did not exceed the accuracy stated by the manufacturer. It shows that colour and type of surface do not influence significantly the RL EDMs, especially on longer distances.
3. Measurements to EPS (material of low density) showed that density significantly influence RL EDM accuracy: the lower the material density, the lower the accuracy. Therefore, reflectorless measurements should be performed cautiously with regard to materials of low density.
4. Errors caused by different types of reflecting surfaces are most significant in the case of short-distance measurements (e.g., 5 m). With increasing distance, the relative error decreased.
5. The accuracy of RL EDMs should be thoroughly investigated in the future, especially with regard to long distances and materials of low density.

## Figures and Tables

**Table 1:** The list of tested materials.

1	Paper, yellow, gloss	28	Ceramics, grey, gloss
2	Paper, yellow, mat	29	Ceramics, grey, mat
3	Paper, orange, gloss	30	Ceramics, black, gloss
4	Paper, orange, mat	31	Ceramics, black, mat
5	Paper, red, gloss	32	EPS *, thickness: 1.7 cm
6	Paper, red, mat	33	EPS, thickness: 3 cm
7	Paper, green, gloss	34	EPS, thickness: 5 cm
8	Paper, green, mat	35	EPS, thickness: 6 cm
9	Paper, blue, gloss	36	EPS, thickness: 8 cm
10	Paper, blue, mat	37	EPS, thickness: 10 cm
11	Paper, brown, gloss	38	EPS, thickness: 15 cm
12	Paper, brown, mat	39	EPS, thickness: 24 cm
13	Paper, grey, gloss	40	Plastic, white, gloss
14	Paper, grey, mat	41	Plastic, white, mat
15	Paper, black, gloss	42	Plastic, red, gloss
16	Paper, black, mat	43	Plastic, blue, gloss
17	Wood, white, gloss	44	Plastic, grey, gloss
18	Wood, white, coarse	45	Plastic, black, gloss
19	Wood, white, very coarse	46	Plastic, black, mat
20	Wood, light brown, gloss	47	Metal, white, gloss
21	Wood, light brown, coarse	48	Metal, white, coarse
22	Wood, dark brown, gloss	49	Metal, grey, gloss
23	Wood, dark brown, coarse	50	Metal, grey, coarse
24	Ceramics, white, gloss	51	Metal, black, gloss
25	Ceramics, white, mat	52	Metal, black, coarse
26	Ceramics, red, gloss	53	Brick, white, coarse
27	Ceramics, red, mat		

* EPS—expanded polystyrene sheets, commonly called Styrofoam.

**Table 2:** Mean values of absolute RL EDM errors on distances: 5, 10 and 20 m.

Type of Material	Parameter	5 m	10 m	20 m
		mm		
Paper	min	−3.4	−3.8	−4.0
	max	−1.4	0.6	−0.8
	mean	−2.27	−1.80	−2.75
Wooden boards	min	−3.4	−3.8	−3.2
	max	2.2	1.8	2.0
	mean	−0.57	−0.94	−0.80
Ceramic tiles	min	−4.2	−3.0	−3.0
	max	1.4	1.2	1.2
	mean	−1.28	−1.00	−1.37
EPS *	min	−1.6	−0.8	−1.8
	max	17.6	14.6	13.4
	mean	8.55	8.30	6.33
Plastic boards	min	−2.8	−1.6	−1.8
	max	3.8	3.8	3.0
	mean	0.46	0.06	0.49
Metal boards	min	−4.6	−2.2	−1.6
	max	4.6	2.6	6.0
	mean	−0.37	0.17	1.17

* EPS—expanded polystyrene sheets, commonly called Styrofoam.

**Table 3:** Mean values of relative RL EDM errors at distances: 5, 10 and 20 m.

Type of Material	Parameter	5 m	10 m	20 m
		%		
Paper	min	−0.07	−0.04	−0.02
	max	−0.03	0.01	0.00
	mean	−0.045	−0.018	−0.014
Wooden boards	min	−0.07	−0.04	−0.02
	max	0.04	0.02	0.01
	mean	−0.011	−0.009	−0.004
Ceramic tiles	min	−0.08	−0.03	−0.02
	max	0.03	0.01	0.01
	mean	−0.026	−0.010	−0.007
EPS *	min	−0.03	−0.01	−0.01
	max	0.35	0.15	0.07
	mean	0.171	0.083	0.032
Plastic boards	min	−0.09	−0.02	−0.01
	max	0.09	0.03	0.03
	mean	−0.014	0.002	0.006
Metal boards	min	−0.03	−0.02	−0.01
	max	0.08	0.04	0.01
	mean	0.020	0.003	0.004

* EPS—expanded polystyrene sheets, commonly called Styrofoam.

**Table 4:** Mean values of absolute RL EDM errors on distances: 5, 10 and 20 m to EPS (expanded polystyrene sheets, commonly called Styrofoam; white). Numbers of samples according to Table 1.

No.	Density	Thickness	5 m	10 m	20 m
	g·cm^3^	mm	mm		
32	0.01	60	12.4	12.6	11.0
33	0.01	100	5.2	6.2	3.6
34	0.01	150	17.6	13.2	12.2
35	0.01	240	14.2	14.6	13.4
36	0.02	17	11.2	7.6	7.0
37	0.02	30	10.6	12.4	7.0
38	0.03	80	−1.6	−0.8	−1.8
39	0.04	50	−1.2	0.6	−1.8
	min		−1.6	−0.8	−1.8
	max		17.6	14.6	13.4
	mean		8.55	8.30	6.33

## Data Availability

Data available at: doi:10.5281/zenodo.5528119.
